# The Outcome of Colonoscopy-Assisted Laparoscopic Wedge Resections (CAL-WR) for Colon Cancer: A Retrospective Cohort Study

**DOI:** 10.3390/cancers17091466

**Published:** 2025-04-27

**Authors:** Robin Glorieux, Julia Hanevelt, Myrtle J. van der Wel, Wouter H. de Vos Tot Nederveen Cappel, Henderik L. van Westreenen

**Affiliations:** 1 Department of Surgery, Isala, Dokter van Heesweg 2, 8025 AB Zwolle, The Netherlands; h.l.van.westreenen@isala.nl; 2 Department of Gastroenterology and Hepatology, Isala, Dokter van Heesweg 2, 8025 AB Zwolle, The Netherlands; j.hanevelt@isala.nl (J.H.); w.h.de.vos@isala.nl (W.H.d.V.T.N.C.); 3 Department of Pathology, Isala, Dokter van Heesweg 2, 8025 AB Zwolle, The Netherlands; m.j.van.der.wel@isala.nl

**Keywords:** colon cancer, local resection, colonoscopy-assisted laparoscopic wedge resections

## Abstract

Local excision is gaining acceptance as standard treatment for T1 colon cancer (CC); however, not all patients are eligible for endoscopic resection. Colonoscopy-assisted laparoscopic wedge resection (CAL-WR) is a relatively new technique that could fill the gap between endoscopic resection and major surgery. The aim of this study was to analyze the oncological safety of CAL-WR for CC. Methods: A retrospective cohort study was performed, including patients that underwent CAL-WR for CC. Exclusion criteria were double tumors, <1 year follow-up, previous other colorectal malignancy, inflammatory bowel disease or synchronous metastases. The primary outcome was disease recurrence and the secondary outcome was overall survival. Results: Fifty-three patients were included; 35 patients were diagnosed with T1 CC. CAL-WR was radical (R0) for all T1 CC in 94.3% and 94.7% for tumors with deep submucosal invasion (sm2-3 Kikuchi). The mean follow-up was 3.3 years (Q1: 2.0; Q3: 4.3) for disease recurrence and 4.2 years (Q1: 2.8; Q3: 5.2) for overall survival. None of the patients with T1 CC had disease recurrence or died due to their malignancy. There were 14 patients with a T2 and 4 patients with a T3 CC, 17/18 patients underwent completion surgery. Three patients with T2 and one with T3 CC developed a locoregional recurrence (peritoneal). One patient with T3 CC developed lung metastases. Two patients with T3 and one with T2 CC died due to their malignancy. Conclusions: This study suggests that CAL-WR is oncologically safe as treatment for T1 CC. The safety of incidental CAL-WR for >T1 CC, followed by completion surgery, remains unclear. Prospective studies are needed to evaluate these results.

## Simple Summary

Local resection of colon cancer is gaining acceptance as standard treatment for low-risk T1 colon cancer. Endoscopic submucosal dissection (ESD) and endoscopic full-thickness resection (eFTR) have their limitations. Lesions with deep submucosal invasion or size larger than 20 mm have a high risk of R1 resection with ESD and eFTR, respectively. It has been shown that colonoscopy-assisted laparoscopic wedge resection (CAL-WR) has no decrease in R0 resection rate in the case of deep submucosal invasion or lesions larger than 30 mm. Therefore, it can possibly expand the options of local resection for T1 CC and thus avoid unnecessary extensive surgery. However, there are currently no studies that describe disease recurrence or overall survival after CAL-WR of CC. This retrospective cohort study focuses on the oncologic results of CAL-WR of colon cancer and can form a platform on which future prospective trials can be built.

## 1. Introduction

Due to the implementation of the population-based colorectal cancer (CRC) surveillance program that started in 2014, the incidence of early-stage colon cancer (CC) is rising in the Netherlands [1,2,3]. T1 CCs are usually resected using advanced local resection techniques rather than conventional oncologic segmental bowel resection. If histological examination shows no high-risk features for lymph node metastases after local resection, the reported risk of recurrent disease is minimal (1%) compared to the overall complication rate (surgical, cardiac, pulmonary, …) of 25% and mortality (1–2%) associated with segmental bowel resection [4,5,6,7]. Subsequently, most patients with low-risk T1 CC are considered curatively treated with local excision alone.

For colonic lesions, three advanced local resection techniques are available. The choice of technique depends on the tumor’s location, size and estimated depth of invasion into the submucosa. Endoscopic submucosal dissection (ESD) can be employed for lesions in both the rectum and colon. However, ESD is technically more challenging in the colon, particularly on the right side, and carries a higher risk of perforation due to the thinner colonic wall [8]. ESD should not be performed if there is a suspicion of deep submucosal invasion, as it often results in an incomplete (R1) resection at the vertical margin [9]. Of importance, the Dutch guidelines changed regarding depth of invasion as a high-risk feature. Sm3 invasion depth (Kikuchi) [10] was considered to be a high-risk feature only until 2022, when the meta-analysis by Zwager et al. proved that the risk of lymphovascular invasion was only 2.6% when no other risk factor is present [11]. This expanded the range of CCs that can be curatively treated with local resection. In the case of deep submucosal invasion, a ‘full-thickness resection’ is recommended, which involves transmural resection of all colonic layers. For lesions smaller than 15 mm, endoscopic full-thickness resection (eFTR) using an over-the-scope device can be performed [12]. However, in larger lesions, eFTR is associated with a substantial higher risk of R1/Rx resection (risk ratio 2.35 per 5 mm increase) and hence should be avoided [12]. Furthermore, there is an increased risk of secondary appendicitis following eFTR when the lesion is situated near the appendiceal orifice [13,14]. If the lesion is located near the appendiceal orifice, the lesion is larger than 15 mm or there is a suspicion of deep invasion, a colonoscopy-assisted laparoscopic wedge resection (CAL-WR) is an alternative treatment modality.

During CAL-WR, the lesion is removed laparoscopically using a linear stapler, while the gastroenterologist assists in localizing the lesion and ensuring luminal patency [15]. This combined endoscopic and laparoscopic procedure was initially introduced as a local resection technique for endoscopically unresectable benign polyps. In a multi-center prospective trial with 118 patients, CAL-WR achieved an R0 resection rate of 91%, 0% leakage and stenosis <1%. Furthermore, there was no difference in R0 resection rate for lesions that are larger than 30 mm, suggesting a clear advantage over eFTR [16]. A possible risk of CAL-WR is the development of stenosis. Therefore, only tumors ≤50% of the circumference are eligible for CAL-WR. In a small series of patients with T1 CC treated with CAL-WR, equal R0 resection rates were reached (88.9%) [17]. Given these excellent outcomes, its short-term and long-term outcomes for T1 CC are currently being investigated prospectively in the LIMERIC-II trial [18].

Of all three aforementioned local resection techniques used in the colon, data regarding long-term oncological outcome are scarce. In this study, we aimed to evaluate the oncological safety of CAL-WR, including rates of locoregional colon cancer recurrence, distant metastases and overall survival among patients with CC following CAL-WR.

## 2. Materials and Methods

A retrospective cohort study was performed. All patients from the LIMERIC-study with histologically confirmed CC were included; these patients were from 9 different hospitals in the Netherlands [16]. Furthermore, all patients (not included in the LIMERIC-study) with histologically confirmed CC who were primarily treated with CAL-WR between 2016 and 2023 in Isala Hospital Zwolle were included. Patients with double tumors, other colorectal malignancy in the last 5 years, less than 1 year of follow-up, a history of inflammatory bowel disease, or those diagnosed with synchronous distant metastases (<3 months after diagnosis) were excluded.

The primary outcome of the study was disease recurrence (locoregional or distant metastases). Locoregional recurrence was defined as recurrence at the stapler line and/or peritoneal metastases. The secondary outcome was overall survival.

Patient characteristics (age, sex, localization of tumor), tumor histology of CAL-WR (risk-features, mucinous subtype) and the histology of completion surgery (if performed) were extracted from the electronic patient records. The resection specimen after CAL-WR and completion surgery were processed according to local protocol: the staple line was removed, and the margins were inked and evaluated for radicality. T1 CC was considered as high-risk if there was any of the following criteria: lymphovascular invasion, poor differentiation, intermediate or high-grade tumor budding (Bd2 or Bd3), and R1 or Rx resection. R1 resection is defined as tumor cells ≤1 mm from the resection margin and Rx is defined as having a nonassessable margin because of the nearby stapler line. During the study period, the Dutch guidelines changed regarding depth of invasion as a high-risk feature. Sm3 invasion depth (Kikuchi) [10] was considered to be a high-risk feature only until 2022, when the meta-analysis by Zwager et al. proved that the risk of lymphovascular invasion was only 2.6% [11]. In this study, T1 tumors were considered as a high-risk tumor if this was concluded at the multidisciplinary meeting at that time, regardless of whether this was based on the recent or old guidelines. Post-operative complications after the CAL-WR and oncologic resection, if performed, were retrieved from the electronic health record and scored according to the Clavien–Dindo classification grade I–V [19]. Follow-up time for disease recurrence (locoregional or distant) was calculated starting from the date of the CAL-WR until the last colonoscopy, staging CT, CEA measurement or diagnosis of locoregional recurrence. Locoregional recurrence was defined as peritoneal metastases or in case when no completion surgery was performed recurrence at the stapler line or local lymph node metastases. Distant metastases were defined as non-locoregional recurrence (liver, lung, retroperitoneal, …). Follow-up time for overall survival was calculated starting from the date of CAL-WR to May 2024, when the data were collected, or until time of death. Statistical Program for the Social Sciences (SPPS) version 24.0 was used to analyze the data and calculate percentages of discrete values as well as the distribution, mean and median of continuous variables. The Shapiro–Wilk and Kolmogorov–Smirnov tests were used to test the normality of continuous variables, respectively, for groups smaller and larger than 50 patients.

## 3. Results

### 3.1. CAL-WR Results

**Figure 1:**
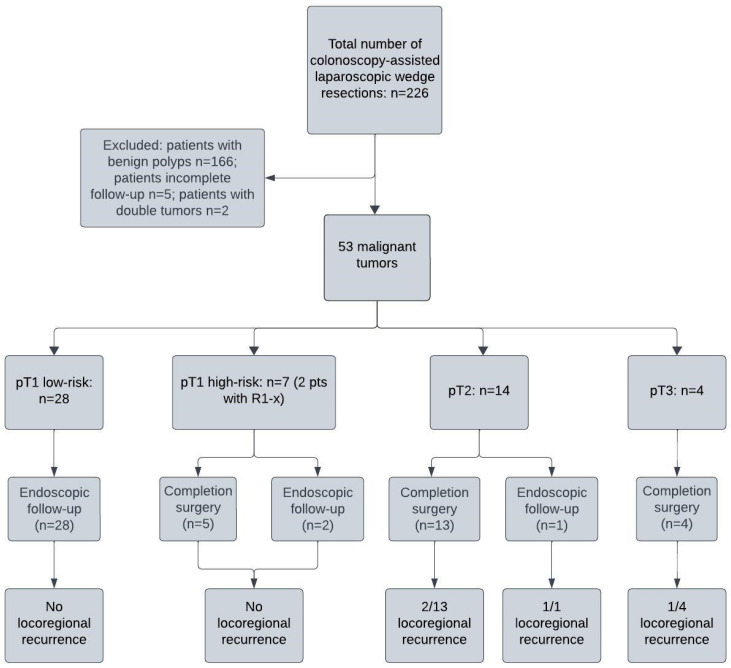
The inclusion and exclusion of patients who had CAL-WR for a colon polyp, the rate of completion surgery or endoscopic follow-up, and the incidence of locoregional recurrence.

Between 2016 and 2023, a total of 226 patients underwent CAL-WR for suspected early colon cancer. After the exclusion of 166 patients with benign polyps, 2 patients with double tumors and 5 patients with incomplete follow-up, 53 patients remained eligible for inclusion (Figure 1). The mean age was 72 years and the majority of patients were male (31 (58%)) (Table 1). Most of the patients were diagnosed with a pT1 carcinoma (66.0%), with seven high-risk pT1 tumors (Table 2). The radical resection (R0) rate for all T1 tumors was 94.3% (33/35) and 94.7% (18/19) for tumors with deep submucosal invasion (sm2-3). Information about differentiation, histology, budding and lymphovascular invasion is summarized in Appendix A. Of note, 11 patients had missing data on tumor budding. Two of these patients had completion surgery because of another risk factor, the other nine patients had no completion surgery.

In almost a third of the cases, after histological examination, the tumor turned out to be a pT2 and, in four cases, a pT3 (Figure 1). CAL-WR was radical (R0) in 64% (9/14) of pT2 tumors and 100% (4/4) of pT3 tumors. For the entire population, an R0 resection with CAL-WR was achieved in 86.8% of the patients. There were no complications after the CAL-WRs.

After the final histological examination and MDT meeting, 25 patients were advised to undergo completion surgery. However, three of these patients refused surgery (Figure 1). Interestingly, none of the patients with R1/Rx resections had any residual tumor in their specimen after completion surgery. Two patients with a pT2 CC and one patient with pT1 CC were diagnosed with lymph node metastases (pN1) in the oncological resection specimen. One patient developed a minor Clavien–Dindo grade 1 (cystitis) complication after completion surgery. There were no technical difficulties during the oncologic resection due to the previously performed CAL-WR.

### 3.2. Locoregional Recurrence, Distant Metastases and Overall Survival

The mean follow-up time for disease recurrence (locoregional recurrence or distant metastases) was 3.3 years (Q1: 2.0; Q3: 4.3). None of the 35 patients, with pT1 CC (both low-risk and high-risk tumors) had locoregional recurrence or distant metastases. Four patients had locoregional recurrence which presented as peritoneal metastases; all four were T2-3 tumors, and two of them also had synchronous distant metastases in the liver (Table 3). One patient was diagnosed with a pT2 mucinous tumor. This patient refused completion surgery even though this was advised in the multidisciplinary meeting. The second and third patient were also diagnosed with a pT2 tumor with, respectively, R1 and Rx resections with CAL-WR. Both of them underwent completion oncological resection; there was no residual tumor nor lymph node metastases. However, the second case had extramural vascular invasion (EMVI). The third case was diagnosed with peritoneal carcinomatosis, after almost 3 years’ follow-up, during a different surgical procedure. The left ovary contained an intestinal-type tumor in which it was impossible to differentiate between an intestinal tract metastasis or primary ovarian cancer (intestinal-type), with immunohistochemical markers showing the expression of CK20 and CDX2. The fourth patient with locoregional recurrence had a pT3 tumor (R0 resection CAL-WR), with no residual tumor nor lymph node metastases after oncological resection. There were no patients with recurrence at the stapler line. One patient with a pT3 tumor developed lung metastases without locoregional recurrence (Table 4).

The mean follow-up time for overall survival was 4.2 years (Q1: 2.8; Q3: 5.2). None of the patients with pT1 tumors died because of their malignancy; three patients died due to non-malignancy-related death. Two patients with pT3 tumors and one with a pT2 tumor died due to CC and four patients died because of non-cancer-related etiologies (Table 3).

## 4. Discussion

CAL-WR was radical (R0) in 94.3% of all T1 tumors and 94.7% for tumors with deep submucosal invasion (sm2-3). None of the patients with a pT1 tumor, including high-risk tumors, had locoregional recurrence or distant metastases during follow up, even after completion surgery. Two patients with high risk T1 tumors refused oncological resection, both were alive without disease recurrence at the end of the follow-up. This seems to confirm our hypothesis that CAL-WR is oncologically safe for the primary local treatment of T1 colon cancer.

In the last two decades, local resection has become common practice for treatment for early colon cancer. Local resection of a low-risk pT1 CC is oncologically safe; there is no difference in disease-specific survival and local recurrence compared with an oncologic bowel resection. Furthermore, prior local endoscopic resection does not increase the risk of post-operative complications after completion surgery [20,21]. For patients with high-risk pT1 CC additional surgical resection is recommended because of the increased risk of lymph node metastases [22,23]. However, in a recent prospective 10-year follow-up study, none of the high-risk T1 tumors, treated only with local resection, had local recurrence. Therefore, the authors suggested that active endoscopic surveillance is a feasible alternative to completion surgery that should be discussed with the patient, especially for patients with comorbidities [24]. This recent literature shows that local resection will become even more important in the treatment for (early) CC. As was mentioned before in the Methods section, the Dutch guidelines differed from some of the other national and international guidelines regarding risk features and, more specifically, for sm3 invasion. The Dutch guidelines changed in 2022, and since then, sm3 invasion alone is not considered a high-risk feature for LN metastases. Only two patients were advised to undergo a completion surgery solely based on the presence of sm3 invasion and seven patients with sm3 were advised endoscopic follow-up. None of these patients developed locoregional recurrence or metastases. Thus, the influence of this changing policy throughout this study and possible difference from other national or international guidelines seems irrelevant.

As mentioned above, there are limitations for ESD and eFTR as an endoscopic technique; CAL-WR could be a technique to fill the armamentarium for the local resection of CC. Our study confirmed this hypothesis, showing an R0 resection rate of 94.3% for all pT1 CC and 94.7% for tumors with deep submucosal invasion (sm2-3). Studies have shown that the R0 resection rate of ESD decreased to approximately 64.7% in lesions with sm3 invasion; therefore, CAL-WR might have a clear advantage for this subgroup of patients [9,25]. In addition, it is known that optical diagnosis is limited in differentiating superficial from deep submucosal invasion and T1 tumors with sm3 invasion from T2 tumors [26,27,28,29]. This demonstrates another advantage of CAL-WR over ESD. The R0 resection rate of eFTR is 88.2% for T1 CC [14]. However, in larger lesions, eFTR is associated with a substantial higher risk of R1/Rx resection [12]. In contrast to eFTR, CAL-WR is not limited by polyp size as long as the polyp does not take more than 50% of the circumference, while eFTR is limited to polyps of maximal 20 mm [16].

There are no data on the effect of quality of life and cost-effectiveness of CAL-WR in this cohort. Evidence for cost-effectiveness and the quality of life impact of CAL-WR on colon cancer is not available in the current literature. However, for benign polyps, it was shown in a retrospective cohort study that CAL-WR is more cost-effective than laparoscopic colectomy with a cost-savings of USD 7103.04 per patient [30]. In addition, it was shown in a prospective study that after CAL-WR for benign polyps, patients perceived no significant impact on the health-related quality of life [31]. Of course, this differs from CAL-WR for malignant colon cancer, because patients might have to undergo subsequent completion surgery. CAL-WR, as a first step in the treatment of CC, should always be a shared decision with the patient. Prospective studies should include outcomes on cost-effectiveness and the effect on quality of life, especially for patients with subsequent completion surgery.

The R0 resection rate of CAL-WR for all patients (pT1-3) was 86.8% and there were no complications after CAL-WR. Completion surgery was performed in 22 of the patients (41.5%); no surgical difficulties due to previous CAL-WR were reported and only one minor complication (cystitis) occurred. Interestingly, none of the tumors with R1/Rx resections had any residual tumor in their specimen. A possible explanation is that the staple line is removed by the pathologist and therefore, the pathology report was not based on the “true” resection margin. This could explain the absence of residual tumor after completion surgery in the patients with R1 resection. In other words, the “true” R0 resection rate in this cohort could be even higher. This is only a hypothesis and not based on prior research.

Almost a third of the patients had a pT2 tumor; this confirms the difficulties of the optical diagnosis of T1-T2 lesions, especially with respect to the depth invasion of the tumor [26,27]. A possible explanation of the high incidence of T2 and T3 tumors is the potential selection bias. CAL-WR is often chosen instead of eFTR or ESD because the tumor is too large or there is a high optical suspicion for deep (submucosal) invasion. Possibly, these tumors were larger and/or particularly more susceptible for deep submucosal invasion and therefore more likely, in fact, to be >T1 CC. It is known that endoscopic differentiation between T1 CC with sm3 and T2 is difficult. Moreover, T2 CC is more likely to be larger than sm3 T1 CC [26]. However, this is only a hypothesis; there were no data to compare the characteristics of all the contemporaneously performed ESDs and eFTRs at the respective centers, nor do we have data on the indication for CAL-WR instead of ESD or eFTR. Koyama et al. proposed a new scoring system to distinguish T1 CC with deep submucosal invasion from T2 CC during colonoscopy [26]. The implementation of this new scoring system might lead to a better selection of cases for CAL-WR. On the other hand, Ichimasa et al. showed that in selected cases, local resection alone should be considered for pT2 tumors [32,33]. CAL-WR could be an essential technique to implement this strategy. However, this was not the subject of interest of this study. Moreover, this is a relatively new approach to the treatment of T2 CCs; it is not included in current guidelines and more research is necessary.

Three out of fourteen (21%) patients with pT2 tumors developed locoregional recurrence (peritoneal metastases). One of these patients had a mucinous CC and refused completion surgery. This is, of course, a suboptimal oncological treatment; incidental CAL-WR of a T2 tumor should always be followed by completion surgery and this could explain the recurrence. In addition, this tumor was a mucinous adenocarcinoma, which is an independent risk factor for peritoneal metastases [34]. The second and third cases were both R1/Rx resections; however, there was no residual tumor after completion surgery. The second case also had EMVI. In a retrospective cohort study by Ravn et al., it was suggested that EMVI is a possible risk factor for peritoneal metastases [35]. Furthermore, EMVI is a known risk factor for the local and systemic recurrence of colorectal cancer [36]. The third case developed peritoneal metastases after nearly three years, though it was not possible to differentiate between a primary ovary or colon tumor with histopathology. Moreover, most patients develop metachronous peritoneal metastases in the first two years after their initial treatment for CC, with a median ranging from 14 to 18 months. This makes it somewhat less likely that the peritoneal metastases originated from the colon in the third case. In a prospective national registry study, the incidence of peritoneal metastases for pT2 CC was <1%, showing that the incidence of 21% (3/14) in our study is high [37]. Of course, CAL-WR of T2 tumors was incidental and therefore, this can give a skewed image. Because the small size of this subgroup in our study and the aforementioned risk factors and ambiguities, no conclusion can be drawn either way. In the current literature, there are no data concerning the prognosis and long-term follow-up for locally resected T2 CC (CAL-WR or eFTR) [32]. R0 resection of T2 tumors with CAL-WR has been reported in two small case series at 100% (8/8) and 62.5% (5/8), respectively [17,38]. The R0 resection rate of eFTR for T2 tumors has only been reported in small retrospective studies, ranging from 52 to 66% [32].

Four of our patients had pT3; all were R0 resections and had completion surgery afterwards. One patient developed peritoneal metastases. T3 CC is a known risk factor for peritoneal metastases [39]. Another patient developed lung metastases after more than 4 years. Both of them died, respectively, two and six years after CAL-WR. It is possible that manipulation of the tumor during CAL-WR caused peritoneal spreading of tumor cells. However, because of the very small sample size, the relation of a previous CAL-WR and the development of peritoneal metastases cannot be proven nor debunked and this should be carefully monitored for this treatment.

To our knowledge, this is the first study that describes the long-term oncological outcomes (locoregional recurrence, distant metastases and overall survival) of CAL-WR. Furthermore, there are no studies in the current literature that report oncologic outcomes after (unintentional) endoscopic resection of >T1 CC. However, the patient cohort was small, and therefore the results should be interpreted with great care and caution. Another limitation is the heterogeneity in the duration of follow-up. Because patients were included between 2016 and 2023, the minimal and maximal follow-up in our population ranged from one to eight years. This could lead to an underestimation of (late) local recurrence. On the other hand, all the cases with locoregional recurrence occurred within three years and a substantial portion of the patients had this duration of follow-up. Another limitation of the study is that 31% of T1 CCs had no records concerning tumor budding, and none of these patients had locoregional recurrence or distal metastasis.

The results of this study show that CAL-WR has good long-term results for the primary treatment of T1 CC. However, because the optical diagnosis of depth invasion in early (T1-T2) CC is suboptimal, the chance of removing T2 or T3 CC is high. Therefore, the oncological safety of removing these tumors with local resection techniques followed by completion surgery should be investigated thoroughly in a prospective study. The relatively high incidence of peritoneal metastasis for the T2 and T3 CCs should be interpreted with great care. Because the small sample size of the T2 and T3 groups could have led to bias, no conclusion can be drawn either way. Our findings demonstrate that the implementation of a new technique for oncologic purposes should be followed with precision and full transparency. The manipulation of the tumor with the linear stapler and the use of a suture close to the tumor can hypothetically cause the spreading of tumor cells in the abdominal cavity. To date, a multi-center, prospective trial (the LIMERIC-II trial) has started in the Netherlands focusing on long-term follow-up after CAL-WR for T1 colon cancer [18]. This multi-center, prospective cohort study will provide further answers about the oncological outcome and safety of CAL-WR of T1 CC; however, as written above, T2 and T3 tumors will inevitably be removed in this study, which will provide us additional information about the oncological safety of the local resection of these tumors.

## 5. Conclusions

The results of our study suggest that CAL-WR has good long-term results for the resection of both low- and high-risk T1 tumors. The safety of (unintentional) CAL-WR of T2 and T3 tumors, followed by completion surgery, remains unclear especially regarding the occurrence of peritoneal metastases.

## Figures and Tables

**Table 1:** Baseline characteristics.

	Total cohort (n = 53); n (%)			
Age (years), mean ± SD	72 (6.7)			
Sex				
○ Male	31	(58.0)		
○ Female	22	(42.0)		
Tumor location				
○ Cecum	10	(18.9)		
○ Ascending colon	13	(24.5)		
○ Hepatic flexure	4	(7.5)		
○ Transverse colon	9	(17.0)		
○ Splenic flexure	1	(1.9)		
○ Descending colon	3	(5.7)		
○ Sigmoid	13	(24.5)		
T-stage			R0 *	
○ pT1	35	(66.0)	33	(94.3)
- sm1	16		15	(94)
- sm2	8		8	(100)
- sm3	11		10	(91)
○ pT2	14	(26.4)	9	(64)
○ pT3	4	(7.5)	4	(100)
Resection status, all tumors				
○ R0	46	(86.8)		
○ R1	5	(9.4)		
○ Rx	2	(3.8)		
Histological tumor type				
○ Adenocarcinoma	43	(81.1)		
○ Mucinous	10	(18.9)		
Complication after CAL-WR	0	(0)		

Abbreviations: R0—radical resection with margin >1 mm; R1—margin of <1 mm; Rx—nonassessable margin because of nearby stapler line. * Percentages are relative for each separate subgroup.

**Table 2:** Completion surgery.

	Cohort (n = x) *; n (%) **	
Completion surgery or endoscopic follow-up		
○ T1 (n = 35)		
- Completion surgery	5	(14.2)
● Histologic high-risk feature	4	(11.4)
● R1/Rx	1	(2.8)
- Endoscopic follow-up		
● Low-risk T1	28	(80.0)
● Histologic high-risk feature	1	(2.8)
● R1/Rx	1	(2.8)
○ T2 (n = 14)		
- Completion surgery - Endoscopic follow-up	13	(93)
	1	(7)
○ T3 (n = 4)		
- Completion surgery	4	(100)
Residual tumor and lymph nodes (n = 22)		
○ No residual tumor, N0	19	(86)
○ No residual tumor, N+	3	(14)
Complication after completion surgery (n = 22)		
○ None	21	(95)
○ Clavien–Dindo grade 1	1	(5)

* The number of the cohort is described in the left column for each subgroup. ** Percentages are relative for each separate subgroup.

**Table 3:** Disease recurrence and overall survival.

	Cohort (n = 53); n (%)	
Locoregional recurrence		
○ Recurrence free	49	(92.5)
○ Peritoneal metastasis	4	(7.6)
○ Recurrence at staple line	0	(0)
Metastasis		
○ Developed metastasis:	3	(5.6)
- Liver	2	(3.8)
- Lung	1	(1.8)
Overall survival		
○ live	46	(86.7)
○ Cancer-related death	3	(5.6)
○ Non-cancer-related death	4	(7.5)

**Table 4:** Cases with locoregional recurrence and/or metastasis.

	Localization Lesion	Histology (CAL-WR)	R0-1-x	Completion Surgery	Residual Tumor	Locoregional Recurrence	Salvage Surgery	Metastases	DFS (Years)	OS (Years)
**Case 1**	Sigmoid	pT2 (mucinous)	R0	Advised (not performed)		Peritoneal metastasis	Not possible PCI > 20	Liver	2.3	3.5 (deceased)
**Case 2**	Sigmoid	pT2	Rx	pT2N0 (EMVI)	No residual tumor	Peritoneal metastasis	HIPEC	None	0.9	5 (alive)
**Case 3**	Descending colon	pT2	R1	pT2N0	No residual tumor	Peritoneal metastasis *	Not possible PCI > 20	Liver	2.7	2.7 (alive)
**Case 4**	Ascending colon	pT3	R0	pT3N0	No residual tumor	Peritoneal metastasis	HIPEC	None	0.8	1.6 (deceased)
**Case 5**	Sigmoid	pT3	R0	pT3N0	No residual tumor	None		Lung	4.3	6.3 (deceased)

Abbreviations: CAL-WR: colonoscopy-assisted laparoscopic wedge resection; EMVI: extramural vascular invasion; PCI: peritoneal cancer index; HIPEC: hyperthermic intraperitoneal chemotherapy; DFS: disease-free survival; OS: overall survival;. * Unclear if peritoneal metastasis originated from ovarian cancer or colon cancer.

## Data Availability

Data can be obtained after reasonable request to the corresponding author.

## Abbreviations

The following abbreviations are used in this manuscript:

CRC	Colorectal cancer
CC	Colon cancer
R1	Tumor cells ≤1 mm from the resection margin
Rx	Uncertain if radical resection
ESD	Endoscopic submucosal dissection
eFTR	Endoscopic full-thickness resection
CAL-WR	Colonoscopy-assisted laparoscopic wedge resection
Bd2 -Bd3	Intermediate- or high-grade budding
SPSS	Statistical Program for the Social Sciences
EMVI	Extramural vascular invasion

## Appendix A

**Table A1:** Post-operative descriptive statistics of all patients with pT1 colon cancer.

	Cohort (n = 35), n (%)			
Submucosal invasion depth			R0 *	
○ sm1	16	(45.8)	15	(94)
○ sm2	8	(22.8)	8	(100)
○ sm3	11	(31.4)	10	(91)
Resection status of al pT1 CC				
○ R0	33	(94.3)		
○ R1	2	(5.7)		
○ Rx	0	(0)		
Degree of tumor budding				
○ Low budding	22	(63.0)		
○ Intermediate budding	1	(2.8)		
○ High budding	1	(2.8)		
○ Missing data	11	(31.4)		
Lymphovascular invasion				
○ None	32	(91.4)		
○ Present	3	(8.6)		
Differentiation				
○ Well-differentiated	34	(97.1)		
○ Poorly differentiated	1	(2.9)		
Completion surgery				
○ Advised	7	(20)		
- High-risk features	5	(14.3)		
- R1 or Rx	2	(5.7)		
○ Performed	5	(14.3)		
Residual tumor and lymph node metastases				
○ No residual tumor, N0	4	(11.4)		
○ No residual tumor, N+	1	(2.9)		
Locoregional recurrence	0	(0)		
Distant metastases	0	(0)		
Malignancy-related death	0	(0)		

Abbreviations: CC: colon cancer; R0: radical resection with margin >1 mm; R1: margin of <1 mm; and Rx: nonassessable margin because of nearby stapler line. * Percentages are relative for each separate subgroup.
